# Prime-Boost Vaccination With Covaxin/BBV152 Induces Heightened Systemic Cytokine and Chemokine Responses

**DOI:** 10.3389/fimmu.2021.752397

**Published:** 2021-10-15

**Authors:** Nathella Pavan Kumar, V. V. Banurekha, Girish Kumar C. P., Arul Nancy, C. Padmapriyadarsini, A. Stella Mary, K. R. Uma Devi, Manoj Murhekar, Subash Babu

**Affiliations:** ^1^ Indian Council of Medical Research (ICMR)-National Institute for Research in Tuberculosis, Chennai, India; ^2^ Indian Council of Medical Research-National Institute of Epidemiology, Chennai, India

**Keywords:** covaxin, cytokines, chemokines, SARS-CoV-2, COVID-19, vaccination

## Abstract

Covaxin/BBV152 is a whole virion inactivated SARS-CoV-2 vaccine. The effect of prime-boost vaccination with Covaxin on systemic immune responses is not known. We investigated the effect of Covaxin on the plasma levels of a wide panel of cytokines and chemokines at baseline (M0) and at months 1 (M1), 2 (M2) and 3 (M3) following prime-boost vaccination in healthy volunteers. Our results demonstrate that Covaxin induces enhanced plasma levels of Type 1 cytokines (IFNγ, IL-2, TNFα), Type 2/regulatory cytokines (IL-4, IL-5, IL-10 and IL-13), Type 17 cytokine (IL-17A), other pro-inflammatory cytokines (IL-6, IL-12, IL-1α, IL-1β) and other cytokines (IL-3 and IL-7) but diminished plasma levels of IL-25, IL-33, GM-CSF and Type 1 IFNs. Covaxin also induced enhanced plasma levels of CC chemokine (CCL4) and CXC chemokines (CXCL1, CXCL2 and CX3CL1) but diminished levels of CXCL10. Covaxin vaccination induces enhanced cytokine and chemokine responses as early as month 1, following prime-boost vaccination, indicating robust activation of innate and adaptive immune responses in vaccine recipients.

## Introduction

Covaxin/BBV152 is a whole virion inactivated vaccine formulated with a toll-like receptor ligand adsorbed to alum (1–4). A phase I trial of Covaxin reported an acceptable safety profile and enhanced immune responses. Neutralizing antibody responses remained elevated in all the participants at 3 months after the second vaccination (5). Phase II trial of Covaxin reported enhanced Th1 responses and antibody responses (6). Moreover, animal studies with this vaccine demonstrated significantly high neutralizing antibody and high-antigen binding titers as well as increased severe acute respiratory syndrome coronavirus 2 (SARS-CoV-2) specific IFNγ producing CD4+ T cells. Based on interim efficacy data from the Phase III trial, Covaxin was approved for use in India. Thus far over 340 million doses of the vaccine have been administered.

Multiple studies have shown that long-term innate immune responses can be either increased (*trained immunity*) or down-regulated (*innate immune tolerance*) after certain vaccines or infections (7–9). Since Covaxin is based on whole virus inactivation combined with a toll-like receptor (TLR) agonist and alum as adjuvants, it is to be expected that Covaxin would induce systemic immune responses in vaccine recipients. However, the nature of these systemic immune responses has not been explored. We, therefore, examined the plasma levels of a panel of cytokines and chemokines in vaccine recipients at baseline and at monthly intervals following administration of the prime-boost vaccine regimen.

## Materials and Methods

### Study Population

We enrolled n=44 individuals (**Table 1**) with a median age of 36. We had 31 males and 13 females, their median height was 169 cm and their median weight was 67 kg, among which one individual had a history of contact with COVID-19 positive case. We had 3 individuals with co-morbidities including type 2 diabetes or hypertension. All the vaccinated individuals had no prior history of COVID-19 infection. The demographics of the vaccinated individuals are shown in **Table 1**.

### Study Procedure

The study recruited healthcare professionals and frontline workers who received BBV152/Covaxin (Manufactured by Bharat Biotech, Hyderabad in collaboration with the Indian Council of Medical Research, India) at vaccination centres in Chennai, India between February 2021 and May 2021. All adult participants of more than 18 years of age, who received two doses of BBV152/Covaxin were eligible to participate in this study. The prime dose was administered at baseline or M0 and the booster dose was administered 28± 2 days later or M1. All participants received both doses within the stipulated time points. Blood was drawn at day 0 (baseline, before vaccination) (M0), day 28 ± 2 days post first dose (M1), day 56 ± 2 days post first dose (M2) and day 86 ± 2 days post first dose (M3). The demographics of the study population are shown in **Table 1**.

**Table 1:** Characteristics of the study cohort.

Study Demographics	
Total number of participants	44
Age in years, median (Range)	36 (23 -60)
Gender (Male/Female)	30/14
Heart Rate (Beats/min), median	78
Respiratory Rate (Breaths/min)	19
BP (systolic), median	115
BP (diastolic), median	76
Height (cm), median	169
Weight (kg), median	67
Contact with COVID-19 Case	1
Tuberculosis	0
Diabetes mellitus	1
Hypertension	2
Cardiovascular disease	0
Chronic Obstructive Pulmonary Disease (COPD)	0
Dyslipidaemia	0
Immunological disease	0
Auto-Immune disease	0
BCG Scar	38

### Multiplex Assays

Circulating plasma levels of cytokines and chemokines were measured using the Luminex Human Magnetic Assay kit 45 Plex (R & D systems) using the BioRad Luminex multiplex ELISA platform. The lowest detection limits for cytokines were as follows: IFNγ, 5.7 pg/mL; IL-2, 3.6 pg/mL; TNFα, 12.4 pg/mL; IL-17A, 9 pg/mL; IL-6, 9.0 pg/mL; IL-12, 18.5 pg/mL; IL-1α, 10.6 pg/mL; IL-1β, 3.5 pg/mL; IL-4, 1.1 pg/mL; IL-5, 6.2 pg/mL; IL-10, 32.2 pg/mL; IL-13, 31.8 pg/mL; IL-25, 18.4 pg/mL; IL-33, 13.8 pg/mL; IFNα, 3.9 pg/mL; IFNβ 3.25 pg/mL; IL-3, 17 pg/mL; IL-7, 3.5 pg/mL; GM-CSF, 18.4 pg/mL; IL-1Ra, 11.7 pg/mL. The lowest detection limits for chemokines were as follows: CCL2, 5.9 pg/mL; CCL3, 5.1 pg/mL; CCL4, 103.8 pg/mL; CCL5, 297 pg/mL; CXCL1, 19.1 pg/mL; CXCL2, 21.1; CXCL10, 2.6 pg/mL and CX3CL1, 188 pg/mL.

### Statistical Analysis

Geometric means (GM) were used for measurements of central tendency. Covaxin vaccinated group at day 0 (M0), month 1 (M1), month 2 (M2) and month 3 (M3) groups were analysed using Kruskal Wallis test one-way ANOVA Multiple comparisons were performed using Graph-Pad PRISM Version 9.0. Principal component analysis (PCA) was done using statistical software JMP 14.0 (SAS, Cary, NC, USA).

### Ethics Statement

The study was approved by the Ethics Committee of ICMR-NIRT (NIRT-IEC No:2021007). Informed written consent was received from all study individuals.

## Results

### Covaxin Induces Enhanced Plasma Levels of Type 1, Type 17, and Other Pro-Inflammatory Cytokines

**Figure 1:**
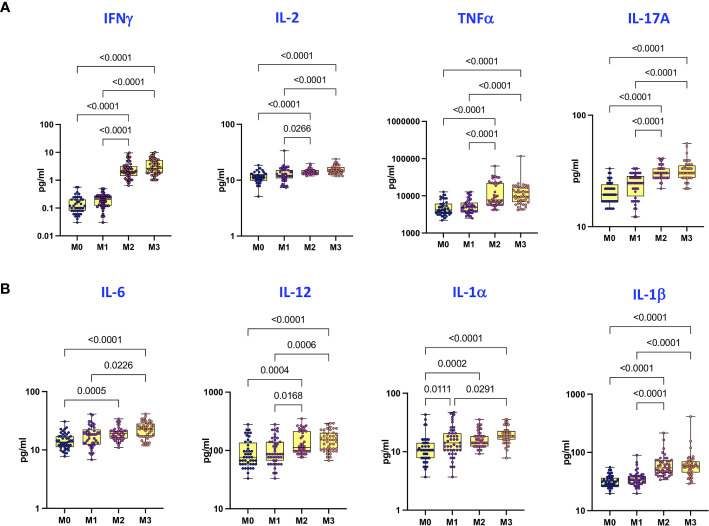
Covaxin induces elevated plasma levels of Type 1, Type 17 and other pro-inflammatory cytokines. **(A)** The plasma levels of Type 1 and Type 17 cytokines in Covaxin vaccinated individuals at baseline [before vaccination, M0] (n = 44) and month 1 following first dose [M1] (n = 44), month 2 following first dose [M2] (n = 44) and month 3 following first dose [M3] (n = 44). **(B)** The plasma levels of other pro-inflammatory cytokines in Covaxin vaccinated individuals at baseline [before vaccination, M0] (n = 44) and month 1 following first dose [M1] (n = 44), month 2 following first dose [M2] (n = 44) and month 3 following first dose [M3] (n = 44). The data are represented as Box and whiskers scatter plots with each circle representing a single individual, p values were calculated using the Kruskal-Wallis test t-test multiple comparisons.

To examine the plasma levels of Type 1, Type 17 and other pro-inflammatory cytokines following Covaxin prime-boost administration, we compared the plasma levels of cytokines at M0, M1, M2 and M3 following the first dose. As shown in **Figure 1A**, plasma levels of Type 1 and Type 17 cytokines, IFNγ, IL-2, TNFα and IL-17A were significantly higher at M2 and M3 compared to M0 and M1. Similarly, as shown in **Figure 1B**, plasma levels of pro-inflammatory cytokines – IL-6, IL-12, IL-1α and IL-1β were significantly higher at M2 and M3 compared to baseline and M1.

### Covaxin Induces Enhanced Plasma Levels of Type 2/Regulatory Cytokines, IL-3 and IL-7 but Diminished Plasma Levels of IL-25, IL-33, IFNβ, GM-CSF, and IL-1Ra

**Figure 2:**
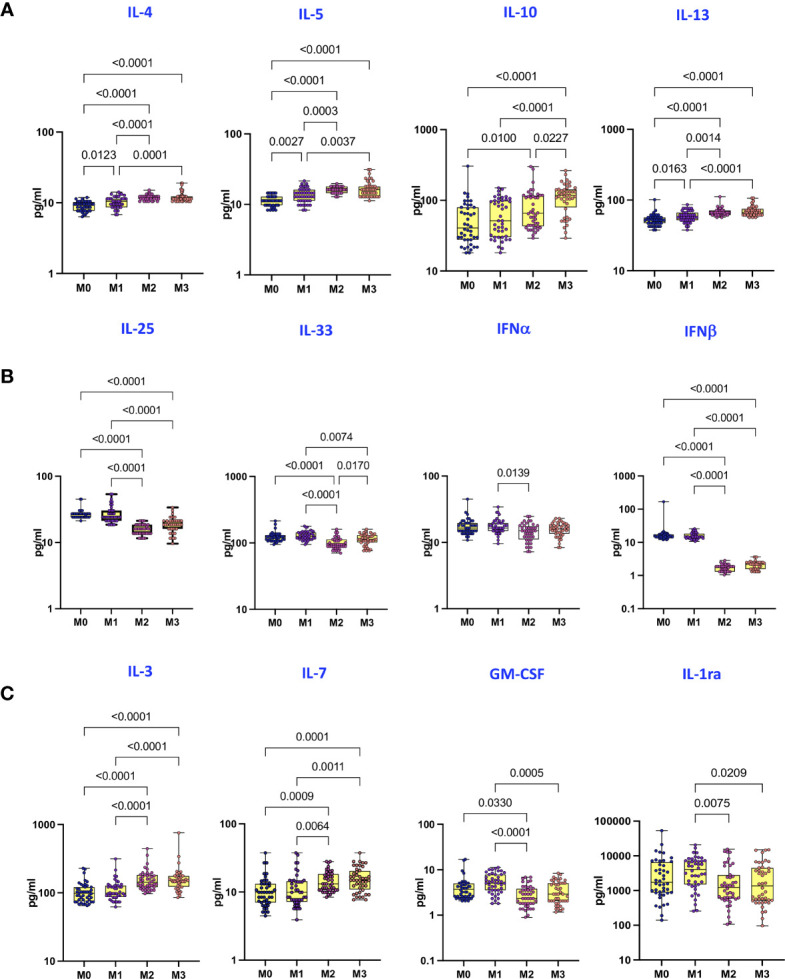
Covaxin induces altered plasma levels of type Type 2/regulatory cytokines, type 1 interferons and other cytokines. **(A)** The plasma levels of Type 2/regulatory cytokines in Covaxin vaccinated individuals at baseline [before vaccination, M0] (n = 44) and month 1 following first dose [M1] (n = 44), month 2 following first dose [M2] (n = 44) and month 3 following first dose [M3] (n = 44). **(B)** The plasma levels of IL-25, IL-33 and type 1 interferons in Covaxin vaccinated individuals at baseline [before vaccination, M0] (n = 44) and month 1 following first dose [M1] (n = 44), month 2 following first dose [M2] (n = 44) and month 3 following first dose [M3] (n = 44). **(C)** The plasma levels of other cytokines in Covaxin vaccinated individuals at baseline [before vaccination, M0] (n = 44) and month 1 following first dose [M1] (n = 44), month 2 following first dose [M2] (n = 44) and month 3 following first dose [M3] (n = 44). The data are represented as Box and whiskers scatter plots with each circle representing a single individual, p values were calculated using the Kruskal-Wallis test t test multiple comparisons.

To examine the plasma levels of Type 2 cytokines and IL-25, IL-33, IFNα and IFNβ following Covaxin prime-boost administration, we compared the plasma levels of cytokines at M0, M1, M2 and M3 following the first dose. As shown in **Figure 2A**, plasma levels of Type 2/regulatory cytokines, IL-4, IL-5, IL-10 and IL-13 were significantly higher at M2 and M3 compared to M0 and M1. In contrast, as shown in **Figure 2B**, plasma levels of IL-25, IL-33 and IFNβ were significantly lower at M2 and M3 compared to M0 and M1. Finally, as shown in **Figure 2C**, plasma levels of IL-3 and IL-7 were significantly higher whereas plasma levels of GM-CSF and IL-Ra were significantly lower at M2 and M3 compared to M0 and M1.

### Covaxin Induces Enhanced Plasma Levels of CCL4 and CXC Chemokines

**Figure 3:**
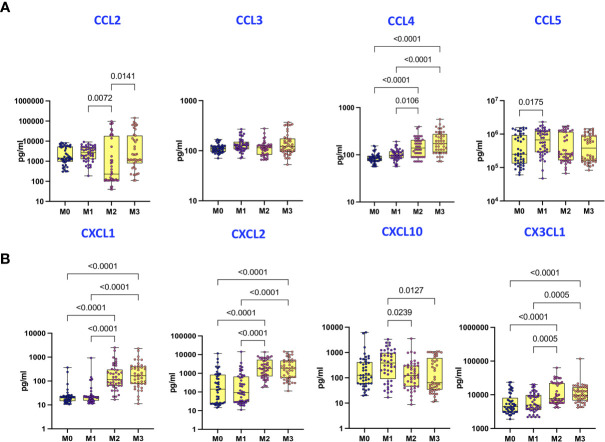
Covaxin induces elevated plasma levels of CC and CXC chemokines. **(A)** The plasma levels of CC chemokines in Covaxin vaccinated individuals at baseline [before vaccination, M0] (n = 44) and month 1 following first dose [M1] (n = 44), month 2 following first dose [M2] (n = 44) and month 3 following first dose [M3] (n = 44). **(B)** The plasma levels of CXC chemokines in Covaxin vaccinated individuals at baseline [before vaccination, M0] (n = 44) and month 1 following first dose [M1] (n = 44), month 2 following first dose [M2] (n = 44) and month 3 following first dose [M3] (n = 44). The data are represented as box and whiskers scatter plots with each circle representing a single individual, p values were calculated using the Kruskal-Wallis test t-test multiple comparisons.

To examine the plasma levels of CC and CXC chemokines following Covaxin prime-boost administration, we compared the plasma levels of chemokines at M0, M1, M2 and M3 following the first dose. As shown in **Figure 3A**, while plasma levels of CCL2 were diminished, plasma levels of CCL4 were significantly increased at M2 and M3 compared to M0 and M1. Similarly, as shown in **Figure 3B**, while plasma levels of CXCL10 were diminished, plasma levels of CXCL1, CXCL2, CX3CL1 were significantly increased at Months 2 and 3 compared to baseline and Month 1.

### Plasma Cytokines and Chemokines Can Distinguish Pre-Vaccinated From Post-Vaccinated Immune Responses

**Figure 4:**
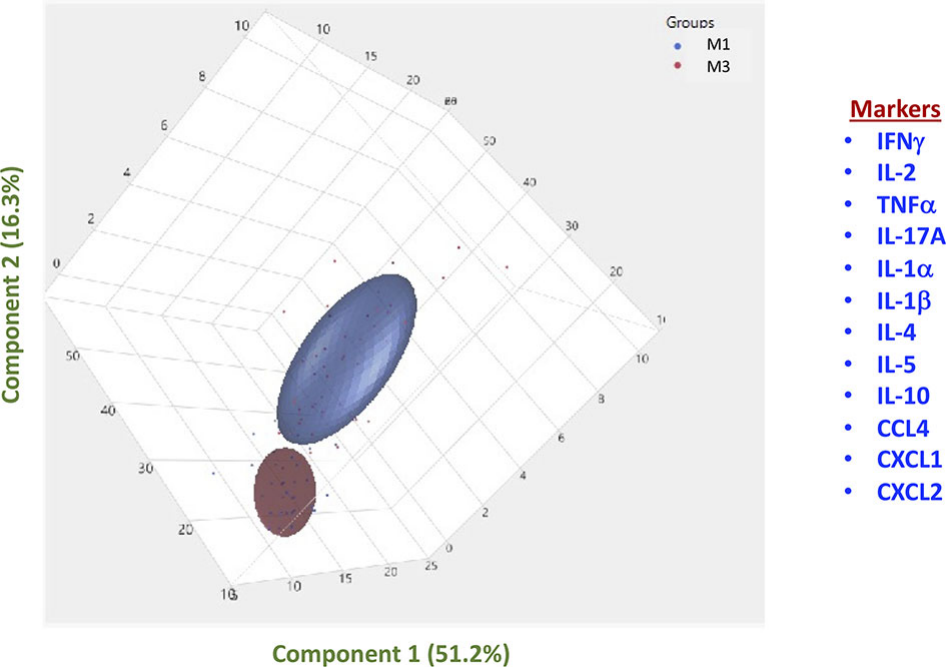
PCA analysis to estimate the discriminatory power of immune markers in Covaxin vaccinated individuals before and after vaccination. PCA (Principal component analysis) plot computing normalized ELISA data from plasma levels of selected cytokines and chemokines in vaccinated individuals at baseline [M0], before vaccination (Coloured in blue) *vs* month 3 [M3] following vaccination (Coloured in red).

We performed PCA (principal component analysis) of IFNγ, IL-2, TNFα, IL-17A, IL-1α, IFNβ, IL-4, IL-5, IL-10, CCL4, CXCL1 and CXCL2 to determine the discriminatory power of plasma cytokines and chemokines in distinguishing pre-vaccinated responses (M0) from the responses induced at M3 following vaccination (**Figure 4**). The two discrete clusters comprising of vaccinated individuals were maintained at the M0 and M3 timepoints. The PCA shows the two principal components of variation, accounting for 51.2% (x-axis) and 16.3% (y-axis). The separation between M0 and M3 based on plasma cytokine levels was also evident on PCA, which markedly demonstrates the ability of these markers to differentiate the systemic immune responses.

### Partial Association Between SARS-CoV-2 Neutralizing Antibodies and Immune Parameters

We wanted to identify correlations between SARS-CoV-2 neutralizing antibodies and immune parameters. We used Spearman’s correlation coefficients to determine the correlation effect and data were visualized by heat map color intensity with variables being ordered by hierarchical clustering. As shown in **Supplementary Figure 1**. A multiparametric matrix correlation plot showed strong correlations between levels of IL-2, IL-17A, IL-4, IL-5 and neutralizing antibodies at M0. However, at M3 we did not observe any correlation between the immune markers and neutralizing antibodies.

### Age and Gender Do Not Have an Effect on the Immune Parameters

We wanted to identify the effect of age and gender on the plasma cytokine and chemokine responses to Covaxin. Median age was determined as 36 and we compared the plasma levels of cytokines and chemokines in vaccinees <36 and >36 at M0 and M3 following the first dose. As shown in **Supplementary Table 1**, plasma levels of cytokines and chemokines are not statistically different between the two age groups. Next to determine the effect of gender, we compared the plasma levels of cytokines and chemokines in men and women at M0 and M3 following the first dose. As shown in **Supplementary Table 2**, plasma levels of cytokines and chemokines are not statistically different between the groups with the exception of CCL3, which was shown to be statistically different between the men and women at Month 0.

## Discussion

Live attenuated vaccines, including BCG (Bacillus Calmette Guerin) and MMR (measles, mumps and rubella) are known to activate innate immune responses in addition to vaccine-specific immune responses, leading to a bystander or off-target effects on the immune system (10–14). The role of COVID vaccines in inducing such an effect remains unexplored. Covaxin is one of the first whole virion inactivated vaccine approved for use against SARS-CoV-2. As such, the immune responses engendered by this vaccine is not completely described, unlike the wealth of data available for the mRNA vaccines against COVID-19 (15–17). Hence, we examined the influence of Covaxin on systemic cytokine and chemokine response, which would reflect the underlying activation of the innate and adaptive immune system by the vaccine. Our data clearly delineate the kinetics of induction of cytokine and chemokine responses in vaccinated individuals. For the most part, the first dose of the vaccine does not induce any significant changes in plasma cytokine and chemokine levels. It is following the second (or booster) dose, that profound changes in the plasma cytokine and chemokine levels are observed. In addition, these alterations persist till the third month following the prime-boost vaccination.

Type 1, Type 17 and pro-inflammatory cytokines (including IL-6, IL-1 and IL-12) are known to play an important role in host immunity to viral infections (18–23). Published studies have reported that, Type 1, Type 17 and pro-inflammatory cytokines may confer long-lasting immune memory against novel coronaviruses (24, 25). In addition, recent studies have also reported that in mRNA-based vaccine (BNT162b2) also elicit better cytokine and chemokine response after 1^st^ and 2^nd^ dose of vaccination in SARS-CoV-2 naive individuals (Bergamaschi C et al. Cell Rep Aug 2021). Therefore, our finding that all of the cytokines examined in the above groups are elevated at a month following booster vaccination suggests the efficient induction of protective cytokine responses following Covaxin vaccination. In addition, while not protective in nature, we also observed induction of Type 2 and regulatory cytokines following prime-boost vaccination in vaccinated individuals. We postulate this to reflect the use of Alum as an adjuvant in the inactivated vaccine since Alum is a known inducer of Type 2 cytokines (26, 27). The effect of Type 2/regulatory cytokine induction in vaccinated individuals needs to be explored further. We also observed the induction of two cytokines with growth-promoting effects – IL-3 and IL-7. While IL-3 is a growth factor for hematopoietic progenitor cells (28), IL-7 is a growth factor for T cells (29), indicating that Covaxin might have off-target effects on progenitor cells as well. Similar to BBV152, other mRNA COVID-19 vaccines BNT162b1 and mRNA-1273 also elicited a profound cytokine response after two doses on vaccination indicating that these vaccines have the potential to protect against COVID-19 infection (30, 31). Interestingly, the only cytokines that were observed to be diminished in our study were IFNβ, GM-CSF and IL-1α. Again, the effect of diminished induction of these particular cytokines needs to examined further. While there are a number of studies in children showing that BCG scar can modulate pro-inflammatory responses, studies have not shown an effect of the BCG scar on cytokine responses in the adult population (32, 33).

CC and the CXC family of chemokines are known to play a vital role in host immunity to viral infections (34–37). These chemokines act by inducing the activation and migration of innate and adaptive immune effectors to the site of infection (38). Our data clearly reflect the elevated induction of CCL4, CXCL1, CXCL2 and CX3CL1, indicating the activation of innate immune cells by Covaxin. Interestingly, we also observed diminished plasma levels of CCL2 and CXCL10 following vaccination, the implications of which are not known currently. Nevertheless, the increased production of certain chemokines following prime-boost vaccination with Covaxin reflects the robust induction of host immunity that is of protective nature by this inactivated vaccine.

Our study has several limitations. We only examined systemic levels of cytokines and chemokines and did not examine vaccine-specific or ligand-specific immune responses. Our findings also suggested that there is no association between age and gender and cytokine responses at M0 and M3, indicating that the induction or reduction of different cytokines or chemokines are not correlated with the either age or gender. Our study is purely descriptive without any mechanistic underpinnings. However, despite these limitations, our study is one of the first, to our knowledge, to describe the induction of systemic cytokine and chemokine responses following COVID-19 vaccination. This data has important implications in terms of understanding the nature of the systemic immune response engendered by COVID-19 vaccines and its effect on bystander immune responses. Biomarkers we have reported in this study can be used as the surrogates of vaccine-induced innate and adaptive protective responses and these biomarkers could also help in the improvement of vaccine efficacy and pertinence

## Data Availability Statement

The raw data supporting the conclusions of this article will be made available by the authors, without undue reservation.

## Ethics Statement

The studies involving human participants were reviewed and approved by Ethics Committee of ICMR-NIRT. The patients/participants provided their written informed consent to participate in this study.

## Author Contributions

SB, CP, and NK designed the study. NK and AN conducted the experiments. NK, KD, GC, and AN acquired data. NK and AN analyzed data. SB, CP, and KD contributed reagents and also revised subsequent drafts of the manuscript. CP, MM, VB, AM, and SM were responsible for the enrolment of the participants and also contributed to acquisition and interpretation of clinical data. SB and NK wrote the manuscript. All authors read and approved the final manuscript.

## Funding

This work was supported by the Indian Council of Medical Research (ICMR).

## Conflict of Interest

The authors declare that the research was conducted in the absence of any commercial or financial relationships that could be construed as a potential conflict of interest.

## Publisher’s Note

All claims expressed in this article are solely those of the authors and do not necessarily represent those of their affiliated organizations, or those of the publisher, the editors and the reviewers. Any product that may be evaluated in this article, or claim that may be made by its manufacturer, is not guaranteed or endorsed by the publisher.

## Supplementary Material

Supplementary Figure 1 Relationship between Immune markers and SARS-CoV-2 neutralizing antibodies. Multiparametric matrix correlation plot of immune markers and SARS-CoV-2 specific antibodies in all individuals at M0 and M3. Spearman’s correlation coefficients are visualized by colour intensity. P values and spearman r values are ordered by hierarchical clustering.
