# Supplementary figures and images for: Prime-Boost Vaccination With Covaxin/BBV152 Induces Heightened Systemic Cytokine and Chemokine Responses
Source: Front Immunol. 2021 Oct 15;12:752397. doi: 10.3389/fimmu.2021.752397 (PMC8554328; doi:10.3389/fimmu.2021.752397)

Sup. Fig. 1A: Month 0 : Nab vs Immune markers

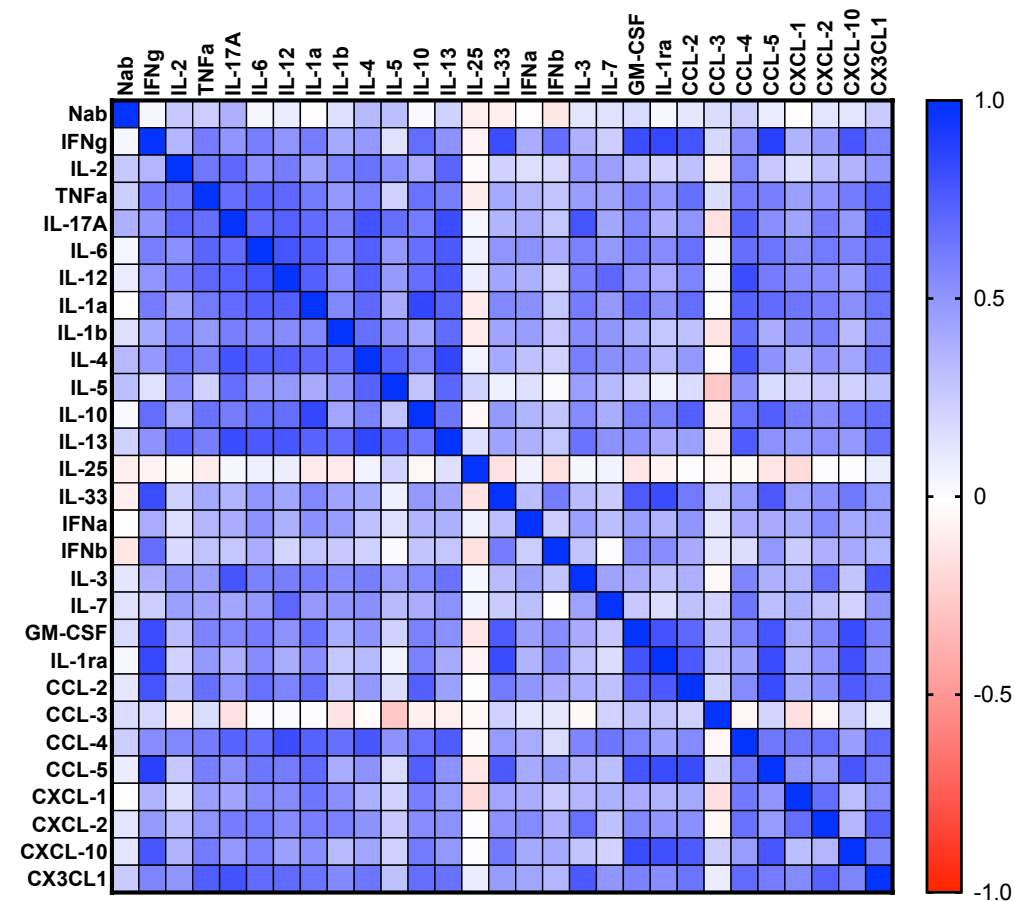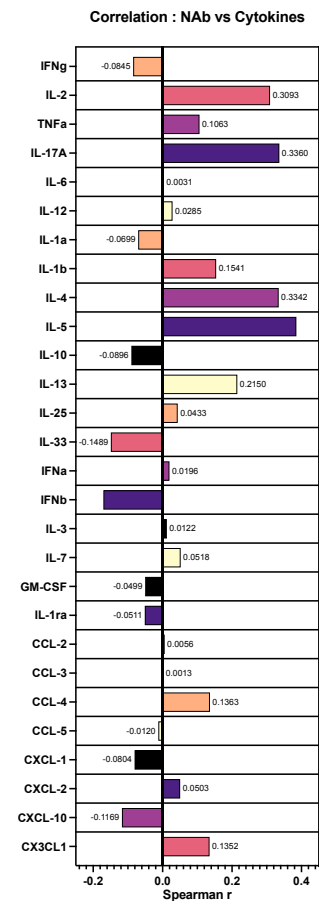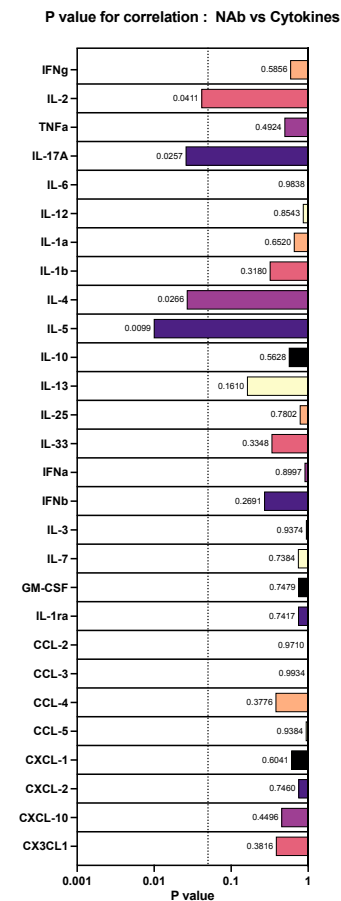

Sup. Fig. 1B: Month 3 : Nab vs Immune markers

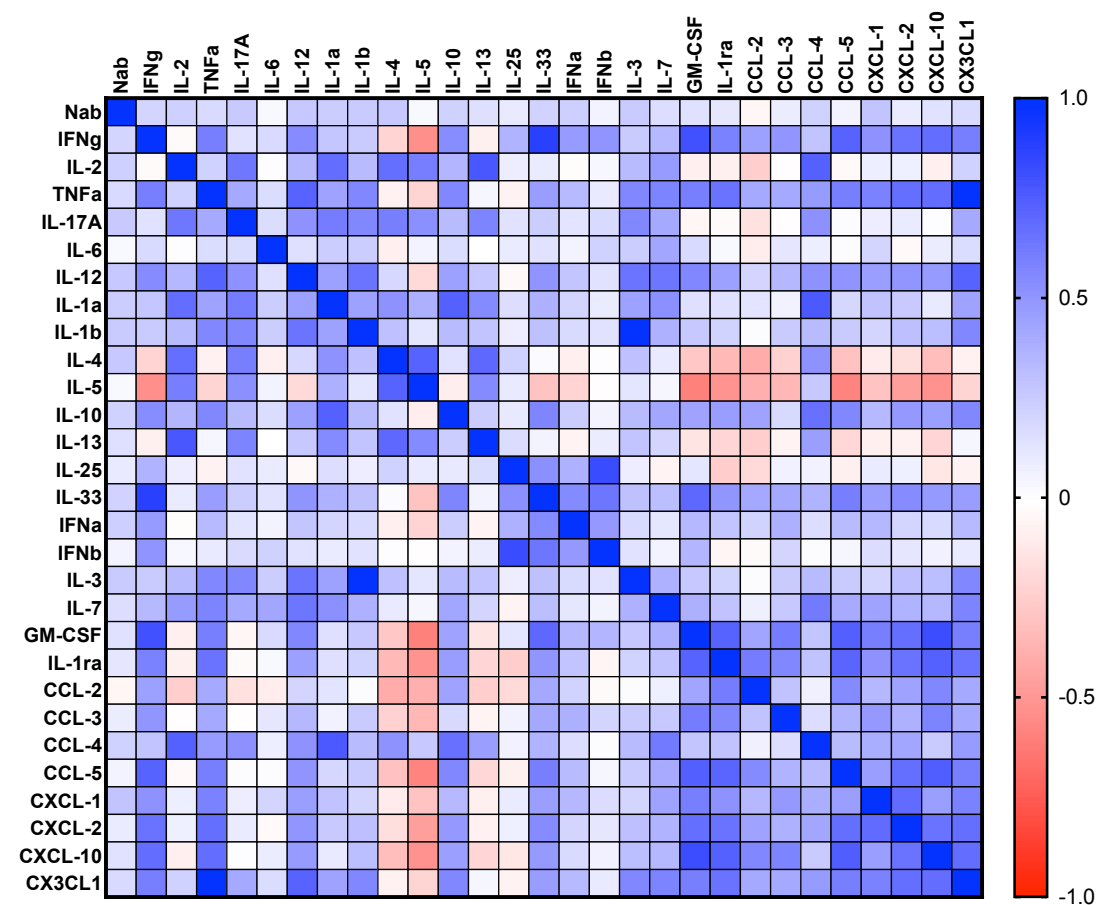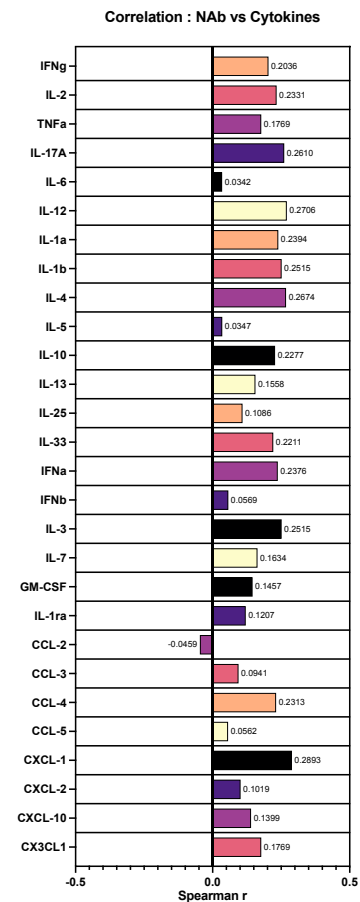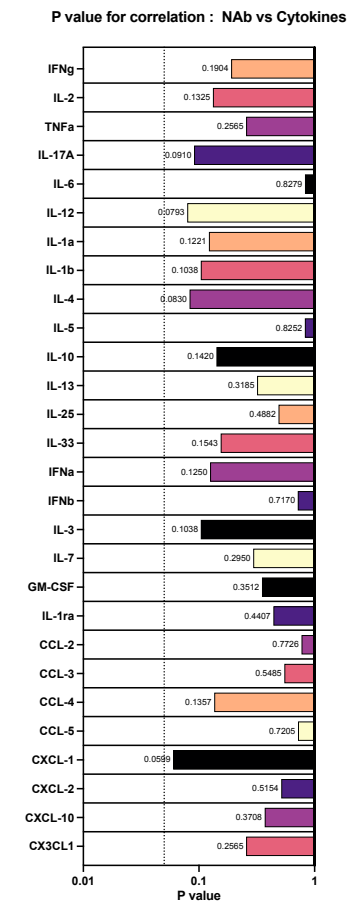

Supplement: Supplementary Figure 1 — Relationship between Immune markers and SARS-CoV-2 neutralizing antibodies. Multiparametric matrix correlation plot of immune markers and SARS-CoV-2 specific antibodies in all individuals at M0 and M3. Spearman’s correlation coefficients are visualized by colour intensity. P values and spearman r values are ordered by hierarchical clustering. [file DataSheet_1.pdf]
