# Supplementary material for: Prime-Boost Vaccination With Covaxin/BBV152 Induces Heightened Systemic Cytokine and Chemokine Responses
Source: Front Immunol. 2021 Oct 15;12:752397. doi: 10.3389/fimmu.2021.752397 (PMC8554328; doi:10.3389/fimmu.2021.752397)
Supplement: Supplementary file 2 [file DataSheet_2.pdf]

Supplementary Table 1 : The plasma levels of cytokines and chemokines were measured in individuals age <36 (n=20) and age >36 (n=24).

	Month 0				Month 3		
**Markers**	**<36**	**>36**	**pValue**	**Markers**	**<36**	**>36**	**pValue**
**IFNγ**	0.1263	0.1199	0.5798	**IFNγ**	2.583	3.15	0.3547
**IL-2**	11.05	11.24	0.7374	**IL-2**	15.62	15.24	0.8176
**TNFα**	4306	4870	0.5721	**TNFα**	10327	10888	0.9953
**IL-17A**	20.25	19.8	0.7259	**IL-17A**	33.65	30.3	0.1349
**IL-6**	12.77	15.26	0.0624	**IL-6**	22.44	20.44	0.3863
**IL-12**	81.37	91.47	0.1972	**IL-12**	137.3	154.2	0.3309
**IL-1α**	10	11.58	0.4826	**IL-1α**	19.64	18.22	0.4717
**IL-1b**	30.71	32.51	0.5051	**IL-1b**	65.89	54.86	0.2545
**IL-4**	8.633	9.257	0.124	**IL-4**	12.52	11.95	0.4529
**IL-5**	11.05	11.34	0.6334	**IL-5**	17.72	15.48	0.0528
**IL-10**	47.25	46.53	0.7666	**IL-10**	108.4	101.7	0.9672
**IL-13**	50.48	53.03	0.5518	**IL-13**	71.95	68.78	0.3924
**IL-25**	25.79	27.37	0.3776	**IL-25**	19.55	17.39	0.2242
**IL-33**	123.1	123.3	0.995	**IL-33**	109.3	113.7	0.4321
**IFNα**	16.26	17.61	0.3534	**IFNα**	16.62	14.61	0.1221
**IFNβ**	15.92	16.61	0.1854	**IFNβ**	2.158	2.006	0.3557
**IL-3**	104.8	96.68	0.5077	**IL-3**	167.7	144.3	0.2545
**IL-7**	8.926	10.71	0.1247	**IL-7**	14.66	15.33	0.668
**GM-CSF**	3.814	3.674	0.6482	**GM-CSF**	2.731	3.319	0.3244
**IL-1ra**	2290	2004	0.7075	**IL-1ra**	1021	1953	0.1199
**CCL-2**	1771	1986	0.5367	**CCL-2**	2343	3579	0.4166
**CCL-3**	109.8	111.5	0.7205	**CCL-3**	115.4	151	0.0984
**CCL-4**	81.14	86.39	0.344	**CCL-4**	183.6	180.8	0.9204
**CCL-5**	335631	314766	0.9518	**CCL-5**	299843	435478	0.2485
**CXCL-1**	5.623	5.651	0.7209	**CXCL-1**	150.6	186.9	0.5692
**CXCL-2**	83.56	147.6	0.4202	**CXCL-2**	299843	435478	0.3592
**CXCL-10**	181.4	151.4	0.6586	**CXCL-10**	90.1	153	0.3942
**CX3CL1**	5956	5093	0.6844	**CX3CL1**	1381	1890	0.9953

*Values appeared are Geometric Mean. P values were calculated using the Mann-Whitney test with Holm's correction for multiple comparisons.

Supplementary Table 2 : Supplementary Table 1 : The plasma levels of cytokines and chemokines were measured in Males (n=30) and Females (n=14)

	Month 0				Month 3		
**Markers**	**Males**	**Females**	**pValue**	**Markers**	**Males**	**Females**	**pValue**
**IFNγ**	0.1191	0.1243	0.8365	**IFNγ**	2.811	3.073	0.7884
**IL-2**	11.12	11.28	0.8065	**IL-2**	15.29	15.65	0.7111
**TNFα**	4728	4419	0.6485	**TNFα**	11012	9889	0.7506
**IL-17A**	19.9	20.17	0.8444	**IL-17A**	31.11	33	0.776
**IL-6**	14.13	14.03	0.9552	**IL-6**	22.6	18.69	0.0694
**IL-12**	88.83	84.04	0.4638	**IL-12**	154.4	131.4	0.2411
**IL-1α**	10.53	11.55	0.5933	**IL-1α**	18.4	19.75	0.6005
**IL-1b**	30.58	34.43	0.0782	**IL-1b**	58.49	61.31	0.6218
**IL-4**	9.026	8.844	0.598	**IL-4**	12.09	12.41	0.7654
**IL-5**	11.1	11.46	0.5739	**IL-5**	16.15	17	0.5548
**IL-10**	43.26	53.81	0.435	**IL-10**	100	114.8	0.5704
**IL-13**	52.1	51.76	0.8546	**IL-13**	69.23	72.1	0.5467
**IL-25**	27.23	25.29	0.057	**IL-25**	17.69	19.66	0.2831
**IL-33**	120.9	127.4	0.2526	**IL-33**	110.6	114.4	0.4556
**IFNα**	17.07	16.92	0.9048	**IFNα**	15.23	15.93	0.5364
**IFNβ**	15.4	18.14	0.7077	**IFNβ**	2.042	2.132	0.5235
**IL-3**	99.25	102.1	0.7502	**IL-3**	152.1	158.1	0.6218
**IL-7**	10.38	9.068	0.2069	**IL-7**	16.17	12.87	0.0519
**GM-CSF**	3.696	3.883	0.6934	**GM-CSF**	3.21	2.737	0.4719
**IL-1ra**	1887	2576	0.3755	**IL-1ra**	1421	1602	0.7985
**CCL-2**	1793	1850	0.9901	**CCL-2**	3288	2401	0.7296
**CCL-3**	117.2	98.38	**0.0014**	**CCL-3**	139.3	124.6	0.473
**CCL-4**	84.2	84.46	0.985	**CCL-4**	175.9	195.7	0.6756
**CCL-5**	298917	365884	0.7181	**CCL-5**	387689	336657	0.7368
**CXCL-1**	4.279	10.84	0.083	**CXCL-1**	168.1	175	0.8276
**CXCL-2**	101.1	152.5	0.5452	**CXCL-2**	1593	1781	0.8325
**CXCL-10**	143.6	220.8	0.333	**CXCL-10**	137.1	94.44	0.4505
**CX3CL1**	5367	5430	0.9233	**CX3CL1**	11012	9889	0.7506

*Values appeared are Geometric Mean. P values were calculated using the Mann-Whitney test with Holm's correction for multiple comparisons.
